# Supplementary material for: A Nomogram Based on Preoperative Lipiodol Deposition after Sequential Retreatment with Transarterial Chemoembolization to Predict Prognoses for Intermediate-Stage Hepatocellular Carcinoma
Source: J Pers Med. 2022 Aug 25;12(9):1375. doi: 10.3390/jpm12091375 (PMC9501090; doi:10.3390/jpm12091375)
Supplement: Supplementary file 1 [file jpm-12-01375-s001.zip › jpm-1810676-supplementary.pdf]

Journal of Personalized Medicine

MDPI

Table S1. Predictors of death for patients with intermediate-stage HCC before third TACE.

Variable	Univariate analysis			Multivariate analysis		
**Before third TACE**	**Hazard ratio**	**95% CI**	***p* value**	**Hazard ratio**	**95% CI**	***p* value**
**Age (yr)** < 60/≥ 60	0.61	0.32-1.34	0.132			
**Sex** male/female	0.65	0.12-1.78	0.342			
**HBsAg status** positive/negative	0.34	0.24-0.89	0.230			
**Child-Pugh** class A/B	1.32	0.23-1.78	0.380			
**Largest tumor size (cm)** < 5/≥ 5	1.23	1.01-3.87	0.023	1.87	0.43-3.28	< 0.001
**Tumor number** ≤3/>3	1.30	1.01-2.89	0.012	1.87	1.02-3.98	< 0.001
**AFP (IU/mL)** < 200/≥ 200	0.62	0.21-1.03	0.098			
**AST (U/L)** < 40/≥ 40	0.54	0.27-0.94	0.081			
**ALT(U/L)** < 40/≥ 40	0.53	0.12-0.78	0.079			
**ALB (g/L)** < 35/≥ 35	1.32	0.76-2.89	0.032	1.45	1.11-1.67	0.067
**Capsule** absent/present	1.78	1.87-3.26	0.012	1.49	0.09-2.76	0.098
**Up to seven criteria** within/beyond	1.65	1.14-1.56	0.043	1.66	1.01-1.87	0.089
**Pre-TACE-3 lipiodol deposition** responder/non-reponder	1.46	0.78-3.88	0.019	1.42	0.45-3.88	< 0.001

BCLC: Barcelona Clinic Liver Cancer; HBsAg: Hepatitis B surface antigen; AST: Aspartate aminotransferase; ALT : Alanine transaminase;. AFP: Alpha fetoprotein; ALB:Albumin.

Table S2. Predictors of death for patients with intermediate-stage HCC before fourth TACE.

Variable	Univariate analysis			Multivariate analysis		
**Before fourth TACE**	**Hazard ratio**	**95% CI**	***p* value**	**Hazard ratio**	**95% CI**	***p* value**
**Age (yr)** < 60/≥ 60	0.42	0.22-1.02	0.821			
**Sex** male/female	0.34	0.24-1.34	0.232			
**HBsAg status** positive/negative	0.44	0.12-0.34	0.451			
**Child-Pugh** class A/B	1.34	0.34-1.34	0.345			
**Largest tumor size (cm)** < 5/≥ 5	1.38	1.33-3.32	0.012	1.26	0.43-2.87	< 0.001
**Tumor number** ≤3/>3	1.34	1.34-3.34	0.023	1.45	1.02-3.36	< 0.001
**AFP (IU/mL)** < 200/≥ 200	0.64	0.23-1.456	0.032			
**AST (U/L)** < 40/≥ 40	0.32	0.12-0.76	0.104			
**ALT(U/L)** < 40/≥ 40	0.33	0.31-0.98	0.321			
**ALB (g/L)** < 35/≥ 35	1.23	0.34-2.78	0.045			
**Capsule** absent/present	1.34	1.32-3.24	0.078			
**Up to seven criteria** within/beyond /	1.32	1.32-1.78	0.032	1.34	1.02-1.89	0.089
**Pre-TACE-4 lipiodol deposition** responder/non-reponder	1.34	0.32-3.45	0.028	1.32	0.23-3.34	0.139

BCLC: Barcelona Clinic Liver Cancer; AST: Aspartate aminotransferase; ALT : Alanine transaminase;AFP: Alpha fetoprotein; ALB:Albumin.
